# Promising use of immune cell‐derived exosomes in the treatment of SARS‐CoV‐2 infections

**DOI:** 10.1002/ctm2.1026

**Published:** 2022-08-21

**Authors:** Murad Alahdal, Eyad Elkord

**Affiliations:** ^1^ Natural and Medical Sciences Research Center University of Nizwa Nizwa Oman; ^2^ Biomedical Research Center, School of Science, Engineering and Environment University of Salford Manchester United Kingdom

**Keywords:** epigenetics, exhaustion, exosomes, immunotherapy, SARS‐CoV‐2

## Abstract

Severe acute respiratory syndrome coronavirus 2 (SARS‐CoV‐2) infection is persistently threatening the lives of thousands of individuals globally. It triggers pulmonary oedema, driving to dyspnoea and lung failure. Viral infectivity of coronavirus disease 2019 (COVID‐19) is a genuine challenge due to the mutagenic genome and mysterious immune‐pathophysiology. Early reports highlighted that extracellular vesicles (exosomes, Exos) work to enhance COVID‐19 progression by mediating viral transmission, replication and mutations. Furthermore, recent studies revealed that Exos derived from immune cells play an essential role in the promotion of immune cell exhaustion by transferring regulatory lncRNAs and miRNAs from exhausted cells to the active cells. Fortunately, there are great chances to modulate the immune functions of Exos towards a sustained repression of COVID‐19. Engineered Exos hold promising immunotherapeutic opportunities for remodelling cytotoxic T cells’ function. Immune cell‐derived Exos may trigger a stable epigenetic repression of viral infectivity, restore functional cytokine‐producing T cells and rebalance immune response in severe infections by inducing functional T regulatory cells (Tregs). This review introduces a view on the current outcomes of immunopathology, and immunotherapeutic applications of immune cell‐derived Exos in COVID‐19, besides new perspectives to develop novel patterns of engineered Exos triggering novel anti‐SARS‐CoV‐2 immune responses.

## Graphical abstract

(1) Immune cell‐derived Exos could be a potential treatment for COVID‐19 by a modulation of immune responses towards virus clearance.

(2) Interaction between SARS‐CoV‐2 and immune cells may induce an epigenetic expression of ICs and exhaustion.

(3) Modified immune cell‐derived Exos could enable an epigenetic stable suppression of viral replication.

(4) Exos have the potential to modulate immune cytotoxic functions towards inhibiting COVID‐19 progression.

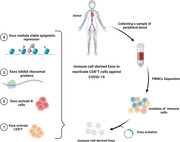

## 1 INTRODUCTION

**FIGURE 1:**
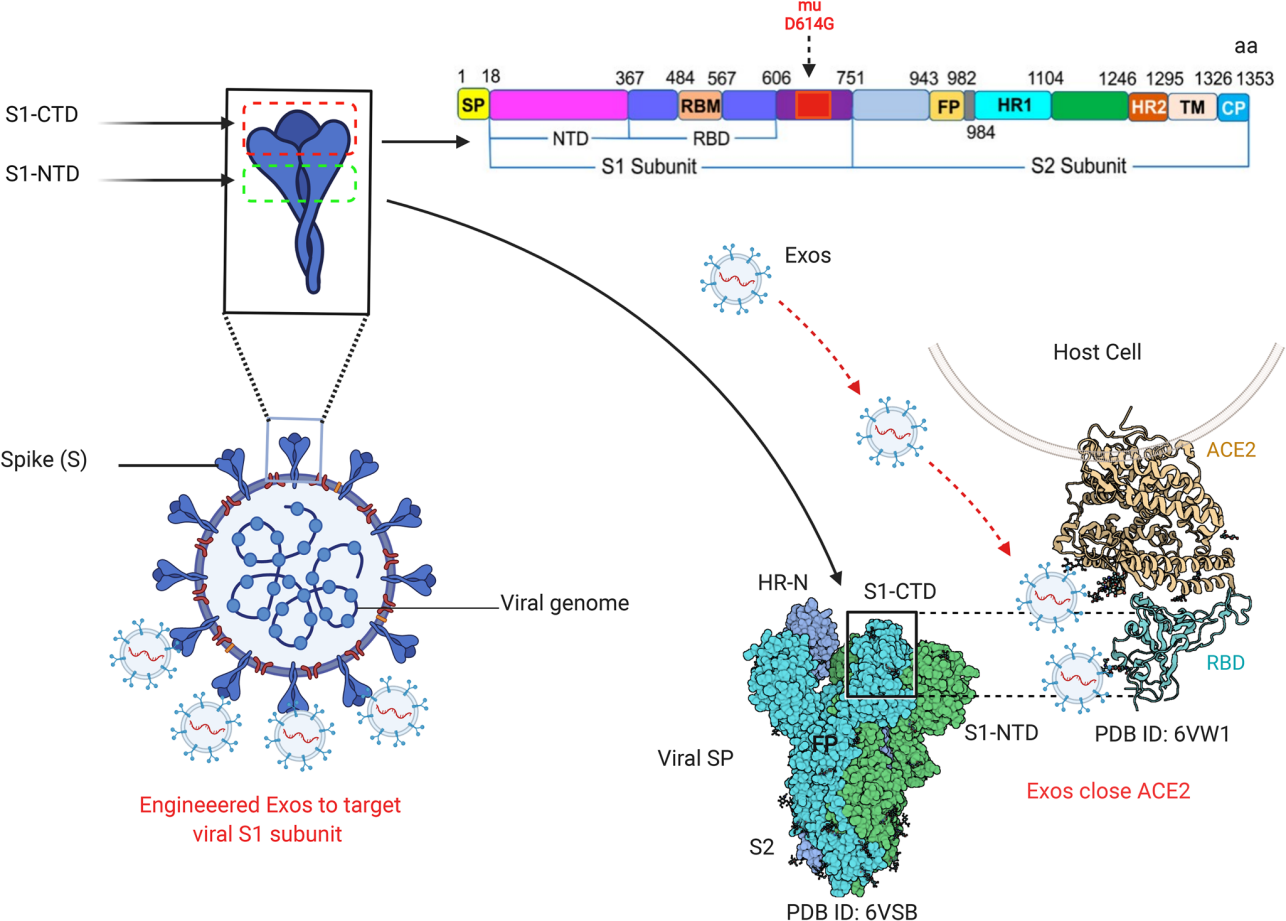
Site of mutations and potential targeting by Exos. Schematic diagram presents the structure of severe acute respiratory syndrome coronavirus 2 (SARS‐CoV‐2)‐SPs, with a focus on mutation positions on the spike proteins (SPs). The S1 subunit is made up of two active parts: S1‐CTDcore, which screens and senses binding active sites; and S1‐CTDmotif, which binds the S1 domain to the active sites of angiotensin‐converting enzyme 2 (ACE2) on the host cell. The potential interaction of Exos with target sites on ACE2 receptors to prevent viral entry

Coronavirus disease 2019 (COVID‐19) is the recently emerged world crisis that caused millions of deaths.^1^, ^2^ This virus causes severe pneumonia, dyspnoea, lymphopenia, cytopenia, hyperferritinemia and cytokine storm, which leads to organ failure called severe acute respiratory syndrome coronavirus 2 (SARS‐CoV‐2).^3^ SARS‐CoV‐2 belongs to the β‐coronavirus (β‐CoV) family which includes SARS‐CoV and other beta CoVs. To date, only seven CoVs can infect humans, SARS‐CoV‐2 is the seventh and the more virulent strain.^4^ As reported, SARS‐CoV‐2, SARS‐CoV and MERS‐CoV can cause acute and severe diseases because they massively infect the lower respiratory tract causing serious pneumonia, whereas OC43, NL63, 229E and HKU1 are associated with moderate infections; they only infect the upper respiratory tract.^5^ In the view of SARS‐CoV crystal structure and analysis of biochemical interaction, SARS‐CoV‐2 targets angiotensin‐converting enzyme 2 (ACE2) receptor by the S1 subunit of spike proteins (SPs). SPs of SARS‐CoV and SARS‐CoV‐2 share 76.5% identity in the amino acid sequences.^6^, ^7^ In the last updates of SARS‐CoV‐2 from 54 sequencing of 5 waves in Iran, different mutations were found in various sections of the genomes. However, the most common mutants were found in the Spike‐D614G, N‐R203K, N‐G204R and NSP12‐P323L.^8^ Further analysis of SARS‐CoV‐2 sequencing confirmed that multiple mutations in the genome concentrated in the open reading frame area and SPs.^9^, ^10^, ^11^ The most important variants and mutations (https://covariants.org/) regarding viral infectivity were found in SPs at the position of 614 (D614G) (see Figure 1). As noticed, mutations in receptor binding protein (RBD) of the SPs enhance the stability of the virus structure and infectivity.^12^ It is obvious that current SARS‐CoV‐2 strains share the same general characteristics as SPs and host cell entrance pathways. When the bound ACE2 receptor is internalized, the cleavage of S protein is initiated by transmembrane serine protease 2 (TMPRSS2), exposing the fusion peptide on the S2 subunit and promoting the entry of SARS‐CoV‐2 inside the cell cytoplasm.^13^

**FIGURE 2:**
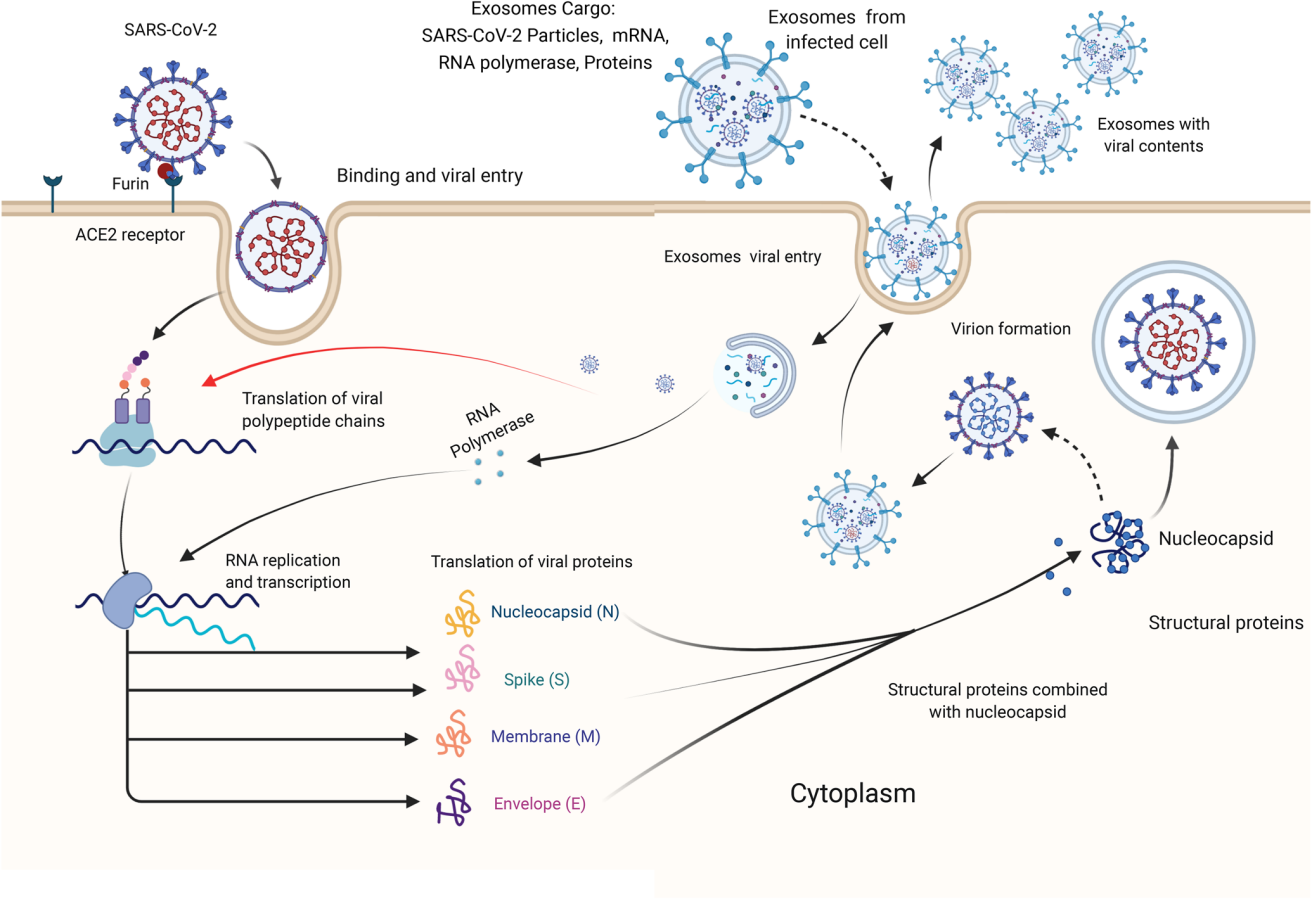
Schematic diagram presents the role of Exos in severe acute respiratory syndrome coronavirus 2 (SARS‐CoV‐2) transmission. SARS‐CoV‐2 particles use furin to induce spike proteins (SPs) cleavage and bind the S1 subunit to the angiotensin‐converting enzyme 2 (ACE2) binding unit to mediate cell entry. SARS‐CoV‐2 begins to replicate after entry, utilizing ribosomes and endoplasmic reticulum to construct virus units. Exos cargo, which includes completed virus units, viral RNA polymerase and uncombined units, is released extracellularly and transmits cargo to healthy cells.

Recently, triggering immune responses to target SARS‐CoV‐2–SPs–ACE2 interaction is a goal for many scientists. However, there are some unclear mechanisms of SPs–ACE2 interaction, and the link with immune cell dysfunction remains to be cleared. Further, the link between new mutations and immune interaction with SARS‐CoV‐2 is still under consideration.^14^ It is believed that the immunomodulation of immune responses could reduce COVID‐19 infectivity through activating specific intracellular pathways that prevent viral replication. The activation of the aryl hydrocarbon receptor by indoleamine 2,3‐dioxygenase 1 (IDO1), which is an immune‐regulatory enzyme causes upregulation of inducible poly ADP‐ribose polymerase (TiPARP) and 2,3,7,8‐tetrachlorodibenzo‐*p*‐dioxin, which enhance SARS‐CoV‐2 replication.^15^ Thus, controlling some intracellular mechanisms may play a role in the suppression of viral replication. This could imply that immune mediators are involved in viral genome mutations by inducing specific intracellular pathways, as recently reported in cancer that mutations in various genes, such as C2, CD163L1 or FCR2A, have been linked to increased or decreased immune cell infiltration, depending on the specific domain altered.^16^ On the other hand, some studies revealed that extracellular vesicles or exosomes (Exos) released from virus‐infected cells essentially contribute to promoting viral transmission and replication,^17^ as presented in Figure 2. Exos released by virus‐infected immune cells could enhance the exhaustion of T‐cell subsets. Recent interactome analyses showed that SARS‐CoV‐2 interacts with Rab proteins which play an essential role in the induction of Exos biogenetic pathways. These findings suggest that Exos released from infected immune cells play an essential role in worsening COVID‐19 infections through regulating immune responses and viral transmission. Fortunately, there are chances to modulate Exos released by immune cells to prevent COVID‐19 complications. Exos released by immune cells such as activated T cells, NK cells and DCs can play a role to block the biogenetic synthesis of viral particles by targeting viral RNA polymerases, leading to viral replication failure. In this review, we discuss the efficiency of immune cell‐derived Exos and the potential opportunities to modify their functions towards alleviation or complete inhibition of COVID‐19 pathogenicity.

## 2 CURRENT IMMUNOTHERAPEUTIC OPTIONS FOR COVID‐19 AND THE POTENTIAL EFFICACY OF EXOS INTERVENTION

Slow rates of virus mutation have been suggested based on the genomic analysis of SARS‐CoV‐2 strains collected from different outbreak regions. Furthermore, all newly reported mutations such as delta, omicron and others have only shown differences in SPs and infectivity, suggesting that immune targeting of viral SPs should be the backbone of virus targeting strategies. The current immunotherapeutic intervention for COVID‐19 infections had no long‐term benefits. In China, the clinical benefits of IL‐6 blockade drugs (Siltuximab; Tocilizumab) manifested a clear benefit in 75% of patients infected with COVID‐19,^18^ but the same drugs in Italy showed only a benefit in 33% of patients.^19^ It is believed that distinct immunological pathways are activated by circulating virus strains, resulting in a different immune‐pathophysiological response that restricts the effectiveness of anti‐IL‐6 antibodies. As a result, using pro‐inflammatory inhibitors may not be the best intervention in cases of severe COVID‐19 infections. Exos could be more effective at reducing the hyperinflammatory response of COVID‐19 because of their power to regulate cellular and humoral responses due to its cargo. Although some scientists thought that available vaccines can be used to treat early COVID‐19 infections by inducing a specific immune response, available data show the low efficacy of those vaccines to induce enough protection.^20^ In the rhesus macaques, SARS‐CoV‐2 inactivated with propiolactone induced promising protective responses against SARS‐CoV‐2 infections.^21^ Vaccinated animals with inactivated SARS‐CoV‐2 showed a rapid decrease in viral loads without any symptoms. However, reinfections among vaccinated individuals were reported, and it did not even help to reduce viral infectivity.^22^

**FIGURE 3:**
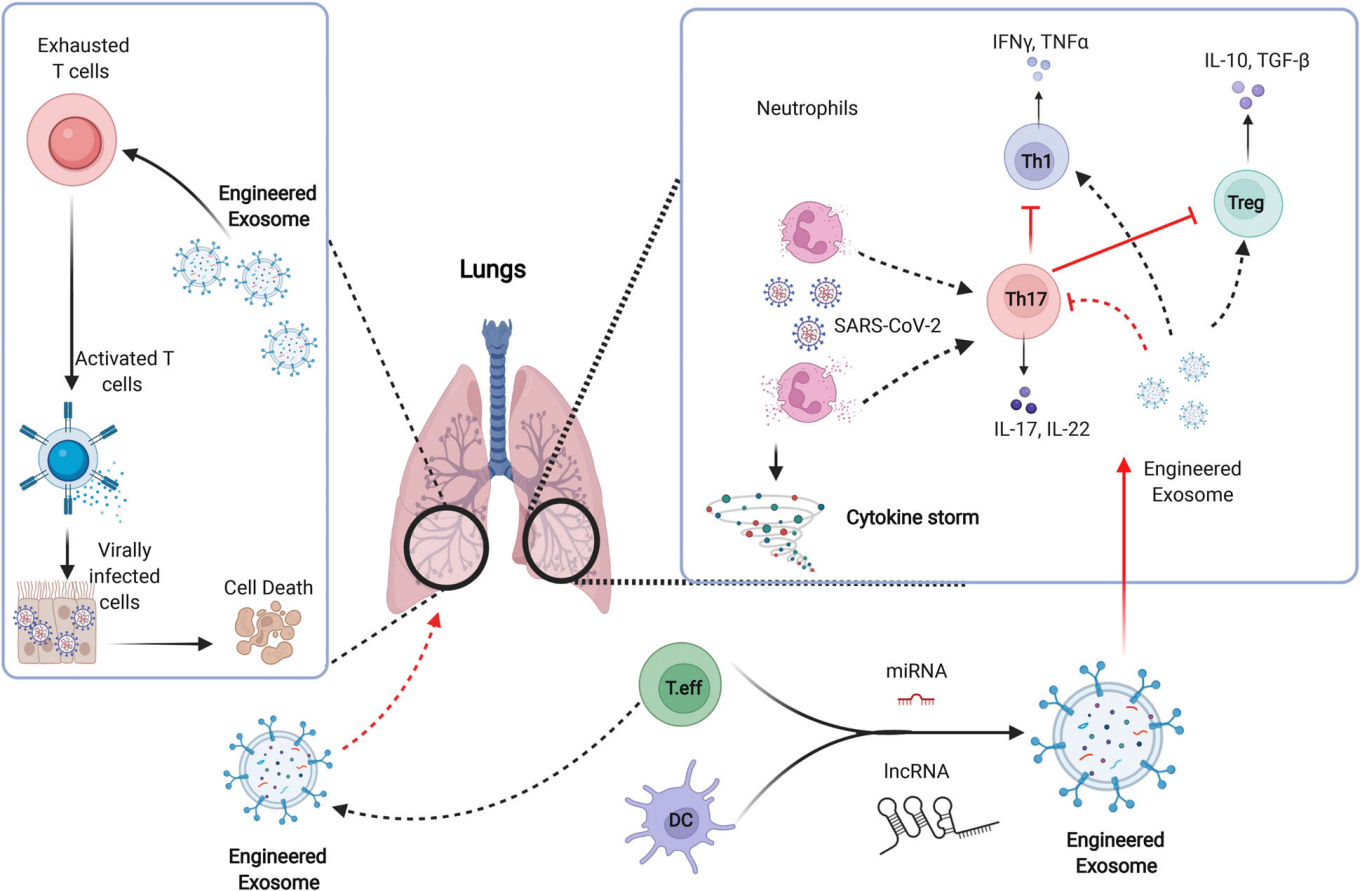
Schematic diagram describes the role of Th17 in Coronavirus disease 2019 (COVID‐19) and the immune modulation effects of Exos. Th17 cells are involved in the progression of COVID‐19 hyperinflammation through inducing repression of Th1 and Tregs. Engineered Exos mediate Th17 cell inhibition and thereby induce functional T effector cell responses and balance the function of active Tregs. Furthermore, Exos enriched with lncRNAs can efficiently induce targeting of virally infected cells by a reactivation of cytotoxic T cells.

In the early stages of COVID‐19 infection, Th17 activation was noticed to be associated with Th1 suppression by raising the level of IL‐17, which mediates the destruction of parenchyma, resulting in respiratory failure. Thus, targeting functional Th17 in COVID‐19 might be particularly crucial in immunomodulation for improving antiviral response. As previously stated, this cell type plays an active role in the induction of hyperinflammation during COVID‐19 infections.^23^ Early reports showed that neutrophils promoted Th17 differentiation in COVID‐19 infections by releasing nitric oxide synthase (NOS).^23^, ^24^ It has been reported that Th17 cells are responsible for Th1 exhaustion and Treg suppression.^25^ This suggests that Th17‐specific targeting may hold therapeutic promises.^25^ On the other hand, Exos have recently received increased attention because, in addition to its capability to promote immunomodulation, it can suppress the immune response by transferring special nucleic acids for targeting signalling pathways.^26^ Further, using Exos released by antigen‐presenting cells (APCs) to present multiple epitopes in viral SP types may work to modulate immune responses early and provide protection against severe infections. In addition, Exos can thus be used to inhibit Th17 proliferation and activity by delivering regulatory miRNAs such as miRAN467b or miRNA146a.^27^, ^28^ This could be an effective strategy to manage the detrimental effect of Th17 in COVID‐19 infections and restore specific viral responses, as presented in Figure 3. A new study reported, using SARS‐CoV‐2‐peptide pools containing structural and functional proteins promoted DCs to strongly support the generation of SARS‐CoV‐2‐specific CD4+ T cells and yielded promising results,^29^ but in other studies, it was noticed that some severe cases did not respond well to this intervention.^30^ We expect that using the whole DCs therapy may interact with other factors that could restrict the capability of DCs to modulate immune responses; however, using Exos derived from SARS‐CoV‐2‐specific CD4+ T cells or DCs activated with peptide pools of SARS‐CoV‐2 SP would be beneficial to trigger rapid viral targeting. The peptide pool library developed from seven active strains of swine influenza in the USA demonstrated good performance to boost immune responses and resist swine influenza subtypes by triggering the production of IFN‐γ.^31^ As previously stated, a unique peptide pool library can be created by combining two components: currently identified viral peptides and predicted peptides that could bind to T‐cell epitopes by interacting with MHC I and MHC II ligands.^32^ Thus, combining currently known peptides from reported SARS‐CoV‐2 subtypes with novel synthetic peptides based on a proteomics analysis of viral peptides that bind to T‐cell epitopes might significantly contribute to the production of a novel peptide pool to trigger anti‐COVID‐19 responses. Using this peptide pool to establish a new pattern of Exos could provide significant resistance against new SARS‐CoV‐2 subtype strains. This pattern of Exos has the potential to induce CD8+ T‐cell responses in a fast manner to the different SARS‐CoV‐2 strains and induce the release of IFN‐γ. The release of IFN‐γ indicates the full activation of T‐cell response, which leads to active clearance of invading viruses. As reported, Exos obtained from plasma of COVID‐19 recovered patients present fragments of viral SPs that enhance significant adaptive immune responses,^33^ suggesting that Exos can present multi‐peptide patterns of different SARS‐CoV‐2 strains through MHC classes I and II.

Furthermore, additional approaches to immunotherapy in COVID‐19 infection can be mediated by inflammatory suppressors such as anti‐IL1β, anti‐TNF‐α and anti‐IFN type 1. These agents demonstrated relative benefits for COVID‐19 treatment in various outbreak areas.^34^ Besides that, the stimulation of IDO1 release may help to reduce hyperinflammatory responses in severe cases, allowing for proper immune targeting of SARS‐CoV‐2. In the same context, the role of Exos in immune regulation was reported for the first time by Raposo et al. in 1996.^35^ Exos treatment of some inflammatory diseases yielded promising results. Exos derived from regulatory mesenchymal stem cells (MSCs) have emerged for treating severe and moderate cases in COVID‐19 immunotherapy clinical trials (Trial NCT04798716 is a registered clinical trial at ClinicalTrials.gov). In phase 1/2 trials, Exos demonstrated promising anti‐inflammatory effects. Until now, the use of immune cell‐derived Exos‐based therapy against COVID‐19 was reported in few studies but demonstrated promising anti‐inflammatory effects, implying that advanced modifications for this type of Exos may reduce cytokine storm and develop more efficient immunotherapies for COVID‐19.^30^ Interestingly, most recent studies regarding the promising effect of Exos in the treatment of COVID‐19 reported unclear mechanisms underlying immune modulation driven by Exos. Few studies paid a focus on Exos‐miRNAs cargo that was reported in the plasma of vaccinated recipients such as miR‐92a‐2‐5p and miR‐148a linked to induction of viral‐specific antibodies,^36^ and miR‐7‐5p, miR‐24‐3p, miR‐145‐5p and miR‐223‐3p linked to the inhibition of viral replication through inhibiting the expression of SPs.^37^ However, no data are available on how these miRNAs work, suggesting the need for further studies. Moreover, the differential studies of circRNAs and lncRNA‐presented 114 circRNAs linked to Exos were differentially expressed in the periphery of COVID‐19 patients, as well as 10 lncRNAs were differentially expressed in circulating Exos.^38^ Overall, these studies highlighted some of the RNAs cargo of Exos in COVID‐19, but underlying mechanisms remain to be elucidated.

## 3 CURRENT SARS‐COV‐2 IMMUNE VACCINES AND POTENTIAL IMPROVEMENTS BY EXOS

Currently, there are several COVID‐19 immune vaccines in clinical trials. SPs of SARS‐CoV‐2 are the most common target in 90% of reported vaccines; LNP‐encapsulated mRNA vaccine that encodes SP (Trial NCT04283461 is a registered clinical trial at ClinicalTrials.gov), DNA plasmid encoding SP delivered by electroporation (Trial NCT04336410 is a registered clinical trial at ClinicalTrials.gov) and Adenovirus type 5 vector that expresses SP (Trial NCT04313127 is a registered clinical trial at ClinicalTrials.gov). Immune vaccines relying on APCs, such as DCs, loaded with lentivirus vectors that produce synthetic minigene based on domains of selected viral proteins (Trials NCT04276896 and NCT04299724 are registered clinical trials at ClinicalTrials.gov), are also being developed. However, a lack of understanding of how immunity develops, host response and pathogen genetic diversity are issues facing these immunological tested vaccines. We believe that using Exos may aid in the development of a promising strategy to reduce viral infectivity and serious complications among infected cases, in addition to inducing long‐term SARS‐CoV‐2 circulating antibodies. Exos can also deliver miRNA and stimulate viral‐specific immune responses by inducing effector T cells and subsequent mechanisms such as cytotoxin release and cell‐death induction.^39^, ^40^ Modulating Exos released from activated T cells may play a novel role in inducing robust anti‐viral antibodies and viral‐specific memory cells.

## 4 POTENTIAL EFFICACY OF EXOS DERIVED FROM IMMUNE CELLS

### 4.1 Tolerogenic dendritic cell‐derived Exos (tolExos)

Exos released by SARS‐CoV‐2‐infected cells can boost intracellular dissemination and transmission due to their simple entry between cells.^41^ This suggests that Exos may play a pivotal role in the enhancement of viral genome replication due to the transfer of contents that may include viral RNA polymerase from infected cells to healthy cells. In contrast, Exos derived from MSC secretome showed significant inhibition to viral replication, reduced cytokine storm, as well as enhanced lung injury repair in COVID‐19.^42^ Exos can mediate a variety of extracellular and intracellular actions. As previously reported, Exos released by tolerogenic dendritic cells (tolDCs) enhance anti‐inflammatory responses by stimulating the proliferation of functional Foxp3^+^ Treg cells.^43^, ^44^ Furthermore, tolDCs‐derived Exos (tolExos) can be loaded with miR‐491, miR‐323 and miR‐654 to inhibit the replication of SARS‐CoV‐2, as these miRNAs demonstrated a promising inhibition of influenza virus replication by blocking conserved regions in viral polymerase basic protein 1 gene.^45^ In COVID‐19, miRNAs that bind to the SARS‐CoV‐2 genome and inhibit post‐transcriptional expressions such as miR‐20b‐5p, miR‐5197‐3p, miR‐17‐5p and miRNA 7114‐5p^46^ are highly recommended to be loaded on tolExos that could inhibit SARS‐CoV‐2 genome replication as well as reducing hyperinflammatory responses. This strategy could contribute to rebalancing immune status and promote quick viral clearance and recovery. New reports indicated that ribosomal proteins (RPs) could be promising targets to block the replication of SARS‐CoV‐2 in host cells and to stimulate immune targeting of infected cells.^47^ However, reports did not establish any clear strategies to exploit this tool. Hence, we suggest that tolExos can be directed towards targeting RPs to inhibit viral genome replication. On the other hand, circulating ACE2^+^ Exos isolated from patients recovered from COVID‐19 can block the binding of SPs domains, leading to preventing SARS‐CoV‐2 transmission.^41^, ^48^ This is encouraging to investigate the possibility of engineered tolExos to target ACE2 binding domains on the SPs for treating severe COVID‐19. Another important point is that COVID‐19 infections increase the damage of alveolar epithelial cells, leading to acute lung injury, which raises secondary infections that increase the risk of dyspnoea. As previously reported, the use of regulatory MSC‐derived tolExos to repair lung injury alleviated the symptoms of inflammation in COVID‐19 patients.^49^ This type of immune intervention is currently being tested in clinical trials (Trial NCT04798716 is a registered clinical trial at ClinicalTrials.gov). It demonstrated promising results in the treatment of lung injury.^50^, ^51^ Furthermore, it is expected that using tolExos loaded with lung tissue repair enhancers such as fibroblast growth factor 7 may provide a novel strategy to cure COVID‐19 by inhibiting hyperinflammation and promoting lung tissue repair.

### 4.2 Exos derived from SARS‐CoV‐2‐specific T cells

Exos derived from mild COVID‐19 cases can stimulate an effective immune response and result in favourable treatment outcomes.^33^ These Exos were obtained from COVID‐19 patients’ plasma and contain the SARS‐CoV‐2 genome, proteins, peptides and other viral‐related components that would be an optimized therapeutic tactic for COVID‐19 patients.^52^ Current clinical trials are focusing on the potential efficacy of Exos derived from virus‐specific T cells (VSTs) for inhibiting infectivity of new strains and releasing sufficient levels of virus‐specific antibodies. As noticed, the use of COVID‐19‐specific T‐cell‐derived Exos (CSTC‐Exo) (trial NCT04389385 is a registered clinical trial at ClinicalTrials.gov) seems promising to reduce death risks of COVID‐19. CSTC‐Exo is used to treat the early stages of the pulmonary disease to control disease progression, but it is planned to be used in the clinic for the treatment of severe cases. Although Exos enhance virus transmission, it can also enhance the expression of CD9 and ACE2 in uninfected cells, which facilitate viral docking and recipient cell susceptibility.^17^, ^53^, ^54^ We believe that modifying VSTs‐derived Exos to target ACE2 mRNA can play a role in the downregulation of ACE2 expression. VSTs‐Exos loaded with miRNAs targeting ACE2 mRNA will directly inhibit the expression of ACE2 leading to prevent viral entry. Furthermore, virus‐derived peptides in Exos from B cells can stimulate B cells to perform dual functions; releasing anti‐viral antibodies and co‐stimulating cytotoxic T cells.^55^ Importantly, the previous research found that Exos derived from IL‐2‐activated T cells effectively reactivate resting T cells against HIV infections.^56^ As a result, we believe that Exos derived from SARS‐CoV‐2‐specific T cells could reactivate resting T cells, restore the function of exhausted T cells and stimulate B‐cell responses because of their special cargo (RNAs, proteins) that can induce cytokine release and immune response activation.

## 5 PROMISING APPLICATIONS FOR EXOS DERIVED FROM IMMUNE CELLS

### 5.1 The role of Exo in restoring functional T cells in COVID‐19

In the last few years, Exos derived from T cells and NK cells demonstrated remarkable potency in triggering active immune responses in autoimmune diseases and viral infections.^57^ As previously stated, the addition of IL‐12 or calcium (CA+) can stimulate the release of Exos from T cells by activating calcium‐dependent mechanisms.^58^, ^59^ T‐cell‐derived Exos, particularly those derived from active cytotoxic T cells, may contain Fas and Apo ligands; the enrichment of these ligands may induce self‐death in virus‐infected cells. Moreover, T‐cell‐derived Exos contain mylins, lymphocyte proteins, ceramides and tetraspanins, which can mediate the targeting of COVID‐19‐infected cells.^60^ Recently, it has been reported that Exos released from active T cells significantly promote the activation of CD3+ T cells, including cytotoxic CD8+ T cells.^59^ It showed superiority to activate resting T cells and enhance the differentiation of effector phenotype and induce the proliferation of T cells in HIV infections through removing tRNA fragments.^61^ Moreover, Exos released from CAR‐T cells showed promising effects to promote anti‐tumour‐immune responses.^62^ However, not enough data are available about the use of Exos to restore the function of exhausted T cells in COVID‐19 infections. A new study revealed that Exos derived from exhausted CD8+ T cells significantly impaired the function of non‐exhausted CD8+ T cells.^63^ Exhausted functional CD8+ T cells may secrete large amounts of Exos, which can be taken up by active CD8+ T cells, as reported in cancer.^63^ These Exos mediate the inhibition of proliferation (Ki67), cell activation (CD69) and cytokine release in recipient cells through transferring IncRNA.^63^ Microarray analysis revealed that 257 lncRNAs were differentially expressed in Exos derived from exhausted CD8+ T cells and non‐exhausted CD8+ T cells. Significant differential expression was noticed in lnc‐hsfy2‐2:3, lnc‐sumf2‐8:1 and RP11‐264J4.8, which regulate the main cellular functions.^63^ Functional enrichment analysis revealed that these lncRNAs actively regulate the diverse processes of CD8+ T cell activity, such as metabolism, gene expression, biosynthetic process and exhaustion, suggesting that Exos derived from active cytotoxic T cells harbour active lncRNAs that could play a role in restoring functions in exhausted T cells, as presented in Figure 3. Moreover, as reported by Yin et al., Exos enriched with miR‐146a‐5p promoted the M2 polarization of macrophages and the exhaustion of T cells.^64^ The inhibition of exosomal miR‐146a‐5p using transcription factor Sal‐like protein‐4 (SALL4) significantly reduced the expression of inhibitory receptors on T cells and reversed T‐cell exhaustion.^64^ This suggests that employing Exos to restore functional cytotoxic T cells in COVID‐19 could be a promising strategy for restoring antiviral immune responses.

### 5.2 Exos can mediate epigenetic modification to alleviate COVID‐19 infectivity

**FIGURE 4:**
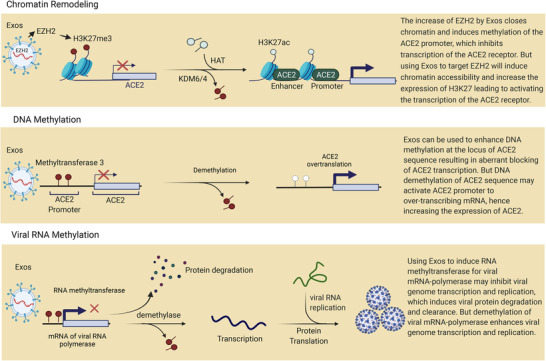
A diagram depicts epigenetic changes in host cell receptors and viral RNA polymerase. The first section demonstrates that Exos enriched in EZH2 mediate chromatin methylation to reduce angiotensin‐converting enzyme 2 (ACE2) expression by activating H3K27me3. The second section demonstrates how Exos enriched in methyltransferase 3 mediate DNA methylation to inhibit ACE2 expression. The third section shows Exos enriched with RNA methyltransferase 3 to induce viral mRNA methylation, which inhibits viral RNA polymerase expression and thus prevents viral replication.

The most difficult challenge in severe COVID‐19 infection is the fast infectivity of the virus due to its high replication rate. Anti‐virus drugs that were used to inhibit SARS‐CoV‐2 replication by targeting RNA polymerase, S protein and 3‐chymotrypsin‐like protease (3CLpro) showed no superiority and limited effectiveness in inhibiting virus replication.^65^, ^66^ In addition, there is insufficient clinical evidence to support the efficacy of current anti‐COVID‐19 drugs.^67^ Recently, Exos were considered a tool for genome epigenetic modification. Exos derived from autologous MSCs can be recruited to induce stable repression of viral replication by triggering RNA/DNA methylation, as reported in the inhibition of HIV replication.^68^ Exos released by cells harbouring HIV‐1 promoter targeting ZPAMt proteins successfully triggered DNA methyltransferase‐3 inducing long‐term repression of HIV replication.^68^ Moreover, in multiple myeloma (MM) patients, Exos derived from adipocyte mediated lncRNA m6A methylation by methyltransferase Like 7A (METTL7A), which led to upregulating LOC606724 and SNHG1 genes that inhibited the apoptosis of MM cells.^69^ Histone modification is enriched at genes associated with functions in T cells that can influence their expression. It is possible to modulate T‐cell function epigenetically in COVID‐19 to make them resistant to exhaustion by regulating the transcription of exhausting markers.^70^ It is suggested that Exos released by epigenetically modified T cells can efficiently prevent the exhaustion of active cells, which could be an ideal intervention to treat severe cases of COVID‐19. In this context, a recent study found that the EZH2 promoter regulates the activity of H3K27me3 to mediate chromatin histone methylation, which inhibits ACE2 expression.^71^ Knocking out EZH2 significantly increases the expression of ACE2. As presented in Figure 4, it is possible that enriching MSC‐derived Exos with the EZH2 promoter could efficiently contribute to inducing chromatin remodelling and lowering ACE2 expression, which could help to prevent SARS‐CoV‐2 entry and replication. Moreover, a new study quantified promoter DNA methylation of ACE2 and TMPRSS2 genes compared to mRNA expression in saliva samples of COVID‐19 patients.^72^ They revealed that DNA methylation of ACE2 has a significant positive relation with mRNA expression of ACE2 in COVID‐19 patients. Interestingly, recent studies highlighted noticeable epigenetic activities in the pathophysiology and severity processes of SARS‐CoV‐2 infections upon the regulation of ACE2 expression.^73^ Regulation of ACE2 gene expression depends on the methylation pattern of several promotor CpG isles, which are associated with gender, age and smoking.^74^, ^75^ Hence, DNA methylation for the ACE2 gene can be mediated by Exos enriched with N6‐methyladenosine or methyltransferase‐3 connected with the ACE2 promoter, as presented in Figure 4. Methylation of viral RNA most likely occurs in the host cell cytoplasm, in association with the replication process through different mechanisms such as the addition of methyl groups to the viral genome; transferring a methyl group to viral protein codes; targeting the methylation of viral RNA polymerase.^76^ As shown in Figure 4, we assume that using tissue autologous Exos to deliver methylation mediating enzymes could contribute to inducing methylation of viral RNA polymerase mRNA leading to inhibiting viral replication. Therefore, we propose that employing epigenetic modification by Exos could contribute to alleviating the expression of ACE2 and reducing viral replication.

## 6 BENEFITS AND LIMITATIONS OF USING IMMUNE CELL‐DERIVED EXOS IN THE TREATMENT OF COVID‐19

Exos have numerous advantages that make them an attractive tool compared to antibody therapy and low molecular weight compounds. It is distinguished by its minimal systemic cytotoxicity, high stability and critical capacity to control cell processes through mediating multifunction. It also can permeate tissues and organs efficiently and offer long‐term effects, whereas antibody therapy needs long‐term time to achieve proper modulation, which needs high effort to be produced with high purity which costs money and time. Furthermore, the stability of antibodies, specificity, toxicity and possible contamination are documented challenges. Exos are easily identified and distinguished by size, and certain markers. Exos can be designed for a variety of purposes, including drug delivery, genetic modification, immune response modulation and cellular uptake enhancement. The most significant aspect of Exos is their ability to home at their origin tissue. Exos from liver cells will migrate to the liver, Exos from the brain will migrate to the brain and others, whereas fused antibodies may not have specific localization properties, and side effects were reported. In addition, Exos can be directed towards a specific cell target based on loaded markers or guidance proteins with few doses and high performance, whereas antibodies need several doses and may present cross‐reaction. Furthermore, stem cell‐derived Exos have the potential to drive immune regulation responses and tissue regeneration, which cannot be seen with antibodies and small molecules therapy. Exos emerged in the treatment of many chronic viral infections, inflammatory diseases and serious cancer types.

On the other hand, although immune cell‐derived Exos have been shown as a new generation of micro tools for modulating internal cell environment and downstream signalling, there are some limitations that need to be addressed. The underlying mechanisms by which Exos mediate immune modulation and antivirus functions are still unclear. The available literature did not broaden the investigation of Exos‐related molecular mechanisms. There is an exception, RNAs cargo of Exos was reported in some new studies. However, those reported RNAs were linked to the direct impact of Exos without further details. This limitation is due to two main reasons: The application of Exos in animal studies and human clinical trials was designed to determine the efficacy and safety in the treatment of COVID‐19 regardless of underlying mechanisms; the uncertainty of Exos cargo makes the identifying of the exact functional moderator is difficult. We expect that comprehensive analysis for immune cell‐derived Exos by processing a deep analysis for total proteins and RNAs (noncRNAs, mRNAs and lncRNAs) may contribute to understand the underlying molecular mechanisms of immune cell‐derived Exos.

Furthermore, there is another limitation, the focus was mainly paid to the human studies, except for some examples from animal studies that support the findings in the human. The reason for this missing was that there have been very few animal studies on Exos derived from immune cells for treating COVID‐19 in animal models, and the quality appears not good. We believe that more animal studies with modified immune cell‐derived Exos are needed to investigate deep mechanisms and the major cargo modulating the effects of Exos against serious infections, including COVID‐19. Other limitations regarding the use of Exos in the treatment of COVID‐19 include the risk of uncertain cargo of Exos, including mRNAs, miRNAs, lncRNAs, proteins and other molecules that can cause undesirable responses or induce autoimmune targeting by transporting autoimmune triggers. Thus, removing undesirable cargo before using Exos could improve the specificity of anti‐COVID‐19 Exos. The technique of removing undesirable cargo depends on the target cargo, for example, removing unwanted miRNAs can be performed by enriching specific circRNAs to sponge target miRNAs, and so on. Moreover, due to the risk of immune surveillance loss, the prudent use of immune cell‐derived tolExos is required. Furthermore, modified Exos for improving activation of T‐cell proliferation and functionality may trigger an epigenetic alteration that could induce transcription of some carcinogenic genes, as reported in lymphoma and leukaemia. Furthermore, Exos separation and purification procedures require further development to improve cargo contents and functionality.

## 7 CONCLUSION

SARS‐CoV‐2 is one of the worst pandemics of the twenty‐first century. Multiple mutations and high viral infectivity are significant obstacles. Although the COVID‐19 spreading trend has currently slowed down, new waves are possible. As a result, it is necessary to look for effective therapeutic options. As presented in Table 1, immune‐derived Exos are gaining popularity due to their numerous functions and effects on physiological and molecular interactions driven by immune mechanisms. According to several studies, Exos involve the progression and severity of COVID‐19 infections, which can mediate viral transmission from infected cells to uninfected cells. Furthermore, Exos released by exhausted cytotoxic T cells can mediate active T‐cell exhaustion. Immune cell‐derived Exos, on the other hand, have the potential to be useful in combating SARS‐CoV‐2 infections. Exos derived from DCs or effector T cells are critical in establishing a proper immune response that leads to virus clearance owing to induce viral‐specific full functional CD8+ T cells. Furthermore, Exos functions can be managed to prevent viral mutation and replication. Exos can also play a role in repressing SARS‐CoV‐2 replication and infectivity via epigenetic repression. They can also be used to stimulate COVID‐19‐specific CD4+ T cells and restore the functionality of effector cytotoxic T cells. Moreover, Exos stimulated by a pool of SPs from different strains of SARS‐CoV‐2 might result in a new pattern of anti‐COVID‐19 Exos, which have the ability to resist subtypes of SARS‐CoV‐2. It is proposed that Exos released by immune cells have the power to facilitate multifunctions (1) restoring or reactivating cytotoxic cells such as CD8+ T‐ and NK cells by transferring regulatory lncRNAs, (2) inducing B lymphocytes to release an adequate quantity of SARS‐CoV‐2‐specific antibodies by direct presenting of viral antigens or transferring mRNA of virus‐specific antibodies, (3) targeting RPs in the infected cells, and (4) mediating stable epigenetic repression for viral replication by targeting replication promoter genes in the genome, as presented in the graphical abstract. However, there may be certain limitations to employing immune cell‐derived Exos in COVID‐19 therapy. It may induce inflammasome and enhance hyperinflammatory response causing cytokine storm. Furthermore, long‐term effects of Exos loaded with SARS‐CoV‐2 SPs could induce CD4+ T‐cell dysfunction due to persistent exposure to the viral antigens. Furthermore, although using tolExos can reduce the risk of cytokine storm, it may suppress the immune‐specific response to the viral antigen. Therefore, Exos could be a promising tool for treating serious COVID‐19 infections.

**TABLE 1:** List of Exos derived from immune cells and their applications

**Exos type**	**Potential active cargo**	**Applications/evidence for SARS‐CoV‐2 and non‐SARS‐CoV‐2 viral infections**	**Features**	**Clinical trials**	**References**
Exos from COVID‐19 plasma	Tenascin‐c and fibrinogen‐beta	Induces NLRP3 inflammasome, Caspase‐1 and IL‐1β	Exos have the capability to induce inflammation		^77^
Monocyte‐derived dendritic cells (moDCs)	Hsa‐miR‐155‐5p	Antigen‐specific CD8+ T‐cell activation	Exos loaded with the native Cytomegalovirus peptide (NLV)	Not registered clinical trial phase 1	^78^, ^79^
Exos derived from genetically modified BM‐DC expressing FasL	Fas ligand	Anti‐inflammatory for treating autoimmune diseases such as collagen‐induced arthritis (CIA)	Exos‐FasL targeting T cells to suppress the inflammatory response		^44^
Macrophage‐derived Exos	Hsa‐miR‐155‐5p	NLRP3 receptor‐dependent inflammasome, Toll‐like receptors (TLR) and TNF‐related signalling pathways	Targeting SOS1 gene, SOCS1 gene, β1 integrins		^80^, ^81^
Exos derived from T‐REx‐293 cells	CD24	Suppress the cytokine storm of COVID‐19	Anti‐inflammatory Exos	Phase 1: NCT04747574	A registered clinical trial at ClinicalTrials.gov
Neutrophils‐derived Exos	High levels of arachidonic acid	Interaction between arachidonic acids and COX‐1 to produce thromboxane A2 (TxA2)	Inducing endothelial cells to express intracellular adhesion molecule‐1 (ICAM‐1)		^82^
Mast cell–derived Exos	HSP‐60 and HSC‐70	Stimulate DCs maturation and enhance antigen presentation/activating T‐cell responses by inducing the interaction of OX40L–OX40	Increasing the expression of CD80, CD86, MHCII and CD40		^83^, ^84^
NK cell–derived Exos	CD56, NKG2D, NCRs, perforin and FAS‐L	Maintaining the homeostasis of immune responses by mediating cytotoxicity against high activated immune cells	Overexpressing CD226 (DNAM‐1)		^85^
NK cell–derived Exos	Bat‐3 receptor, NKp30 and loaded with HIV‐antisense protein	Activating CD8+ T lymphocytes	Presenting HIV‐antisense protein	Phase 1: NCT05243381	A registered clinical trial at ClinicalTrials.gov
T‐cell‐derived Exos	IFN‐γ and SARS‐CoV‐2 fragment peptides	Activate virus‐specific T‐cells (VSTs)	CSTC‐Exos	Phase 1: NCT04389385	A registered clinical trial at ClinicalTrials.gov
CD3+ T‐cell‐derived Exo	Tetraspanins, CD9, CD63, CD81, annexins Rab, GTPases and flotillin	Transfer miRNAs from the T cell to the DCs	Modulating DCs function		^86^
Treg‐derived Exos	CD25, CTLA‐4 and CD73	Prevent the proliferation of Th1 and the production of IFN‐γ by transferring microRNA‐155, let‐7b and let‐7d	Suppressing immune response through releasing IL‐10 and TGF‐β1		^87^, ^88^
B‐cell‐derived Exos	CD19, α4β1 integrins, β1 and β2 integrins	Activate follicular dendritic cells (FDCs) through binding with VCAM‐1. Further, they can activate T cells	Activating CD8+ T cells in an antigen‐specific manner		^89^, ^90^

Abbreviations: COVID‐19, coronavirus disease 2019; SARS‐CoV‐2, severe acute respiratory syndrome coronavirus 2.

## CONFLICT OF INTEREST

The authors declare there are no financial or non‐financial conflicts of interest.
